# Pelvic lymph node motion during cone-beam computed tomography guided stereotactic radiotherapy

**DOI:** 10.1016/j.ctro.2024.100794

**Published:** 2024-05-11

**Authors:** J. Janssen, F.H.E. Staal, J.A. Langendijk, S. Both, C.L. Brouwer, S. Aluwini

**Affiliations:** Department of Radiation Oncology, University of Groningen, University Medical Center Groningen, Groningen, the Netherlands

**Keywords:** Stereotactic radiotherapy, Oligometastasis, Lymph nodes, Margins, Target motion

## Abstract

**Background and purpose:**

Stereotactic body radiotherapy (SBRT) is increasingly applied for pelvic lymph node recurrence. Thus far, knowledge on pelvic lymph node motion during CBCT-guided SBRT is lacking and the applied margins vary between institutions. This study evaluated pelvic lymph node motion during CBCT-guided SBRT and assessed the currently applied PTV margins of 3 and 5 mm.

**Material and methods:**

In total, 45 pelvic lymph node metastases were included. One observer delineated 45 GTVs on planning CT, 224 GTVs on pre-fraction and 216 on post-fraction CBCT. The GTV centroid coordinates were derived from all images for inter- and intrafraction motion analysis. Additionally, we assessed the influence of treatment time and lesion location on lesion motion. The expected coverage of a 3-mm and 5-mm PTV margin was assessed using the inclusiveness index for GTVs on pre- and post-fraction CBCT.

**Results:**

Lymph node interfraction motion was limited to 5 mm in 96–97 % of fractions for all translational directions and intrafraction lesion motion was limited to 3 mm in 97–100 % of fractions. Para-rectal lesions (11 %) were associated with significantly larger inter- and intrafraction motion compared to other pelvic locations and treatment duration showed no correlation with lesion motion. The mean (sd) lesion inclusiveness index was 99 % (5 %) for the 5-mm PTV margin and 96 % (9 %) for the 3-mm margin.

**Conclusion:**

Pelvic lymph node motion during CBCT-guided stereotactic radiotherapy was within the widely applied PTV margin of 5 mm, providing an opportunity to reduce this margin for pelvic lymph node SBRT.

## Highlights

• Pelvic lymph node intrafraction motion was limited to 3 mm in 97–100 % of fractions.
• A 3-mm PTV margin was associated with a high mean lesion inclusiveness index of 96 %
• Para-rectal lymph nodes showed increased lesion inter- and intrafraction motion.

## Introduction

Stereotactic body radiotherapy (SBRT) has become an important treatment option for oligometastatic disease, especially in case of recurrent prostate cancer [1], [2]. Several studies reported better outcome of SBRT in case of regional disease, which led to a rapid increase in the application of SBRT to pelvic lymph nodes [3], [4]. In most centres, a treatment schedule of 3 fractions of 10 Gy or 5 fractions of 7 Gy is applied for lymph node SBRT, and planning target volume margins vary between 3 and 5 mm [5], [6], [7], [8]. The high dose per fraction and limited number of fractions used in SBRT obligate margins that preserve the balance between oncological control and normal tissue complications. The traditional approach for margin calculation is based on (conventional) fractionated EBRT and, therefore, less suitable for the stereotactic setting [9]. As a consequence, the currently used margins in SBRT are mostly based on institutional experiences, which can lead to undesirable, wide and inconsistent variations between treatment centres [1], [8], [10].

Important factors potentially leading to a geographical miss of the tumour during treatment are inter- and intrafraction patient and lesion motion. For (pelvic) lymph node SBRT specifically, patient motion has been described for both MRI linear accelerator (MRI-linac) and cone beam computed tomography (CBCT) linac treatment and in both cases, basic immobilization was associated with limited patient motion during treatment [11], [12]. Analyses describing pelvic lymph node lesion motion, however, are scarce. For CBCT based SBRT, lesion interfraction motion has been assessed by reporting organ at risk motion [13]. Another study used repetitive MRI during CBCT-linac treatment to assess pelvic lymph node interfraction motion in patients with primary prostate cancer [14]. As interfraction motion can be largely eliminated in online image-guided SBRT, intrafraction motion (motion during treatment) plays the most substantial role in accurate delivery of SBRT treatment. Thus far, the few analyses available on lymph node intrafraction motion have reported intrafraction motion solely for MRI-linac based SBRT [11], [15]. CBCT-guided SBRT differs largely from MR-guided treatment considering lesion visibility, verification technique and treatment duration [16]. Given that CBCT-guided treatment is still the most used and widely available technique to treat pelvic lymph node recurrence with SBRT worldwide, the evaluation of both inter- and intrafraction lymph node motion on CBCT is crucial to assess accuracy of treatment delivery and evaluation of the optimal margins. With this study we aimed to provide insight in pelvic lymph node motion during stereotactic treatment on CBCT-linac and evaluate the robustness of the currently used PTV margins of 3 and 5 mm. Furthermore, we aimed to identify factors associated with large intrafraction motion during CBCT-guided SBRT.

## Materials and methods

### Patients and lesions

In total, data of 37 patients with prostate cancer included in the ADOPT clinical trial (NCT04302454 [17]) were used to analyse lesion motion. Patients provided written informed consent for the use of their data. Patients were treated between March 2020 and June 2022 with SBRT aimed at pelvic lymph node metastases in 5 fractions of 7 Gy. Lesion localisation within the pelvis was divided into six locations: adjacent to the a. iliaca interna, a. iliaca externa or a. iliaca communis, located within the obturator area, para-rectal region or presacral region. The visibility on CBCT was quantified by a five-point scale rating ranging from very poor to excellent, and a rating of average, good or excellent was required to minimize bias due to inaccurate delineation on CBCT. Hereby, lesions with insufficient visibility (poor to very poor) were excluded from this analysis. Furthermore, lesions with <3 post-fraction CBCTs available were excluded.

### Treatment

The applied gross tumour volume (GTV) to planning target volume (PTV) margin was 5 mm for all lesions conform our institutional protocol. Patients were immobilized using simple immobilization as applied within our centre [12]. Patients were set-up to the isocentre by patient markings as defined during the planning CT (tattoos), and lasers. Treatment planning was conducted using RayStation (RaySearch Laboratories, Stockholm SWE), and consisted of 2 VMAT arcs. Dose constraints for the PTV were V100 % (V3500cGy) > 95 % and V95 % (V3325cGy) ≥ 99 %. During treatment (Elekta Synergy®, Elekta Solutions Ab, Stockholm SWE), online acquired 3D-CBCT images (pre- and post-fraction) were registered to the planning CT using grey value based automatic image registration and a registration mask including the pelvic bone structures adjacent to the PTV (Elekta XVI, Elekta Solutions Ab, Stockholm SWE). The image registration results were translated to couch translations in left–right (LR), anterior-posterior (AP) and superior-inferior (SI) direction. After the grey value based automatic image registration of the pre-fraction CBCT radiotherapy technologists performed a visible check of lesion alignment within the PTV and performed manual corrections if necessary.

### Inter- and intrafraction lesion motion

Planning CT and CBCT data were imported into Mirada DBx 1.2.0 (Mirada Medical Ltd, Oxford UK) and one physician delineated the lymph node GTVs on all planning CT and pre- and post-fraction CBCT images. Thereafter, the corresponding planning CT was matched with the CBCTs using grey value based automatic image registration and the registration mask for translations only. The delineated GTVs were transferred to the planning CT for analysis.

The centroid coordinates of the GTV originating from the planning CT, and pre- and post-fraction CBCT were used to report inter- and intrafraction motion in three translational directions. A negative shift indicated displacement towards the right (LR axis), anterior (AP axis), or superior (SI axis) direction, respectively. Additionally, a three-dimensional vector displacement (3D vector) was reported to assess the overall shift. Rotational lymph node motion was out of scope for the report of lesion motion in this study since the rotational movement of spherical targets (lymph node GTVs) has been reported to have limited influence on coverage [9]. Furthermore, we assessed the association of treatment time, lesion location and lesion volume with lesion inter- and intrafraction motion.

### GTV coverage

GTV coverage was assessed using the inclusiveness index. The inclusiveness index is calculated as the percentage of the GTV that is covered by the PTV (volume of covered GTV divided by total GTV times 100 %) and describes coverage independent of planning technique and planning system. We assessed the inclusiveness index per fraction and per lesion for a 5-mm and 3-mm margin added to the GTV on planning CT. The inclusiveness index per fraction was quantified as the mean value of the pre- and post-fraction inclusiveness indices, and the inclusiveness index per lesion was subsequently quantified as the mean value over the entire treatment course.

For all lesions, we compared the inclusiveness index of the 5-mm PTV with the treatment planning coverage (V3325 cGy (V95%)) to evaluate the conformity between the two parameters.

### Statistics

Statistical analysis was conducted using IBM SPSS Statistics v28. The lesion motion was reported using the mean value along with the standard deviation (sd). The inclusiveness index and treatment planning coverage were presented by the mean of pre- and post-fraction coverage (per fraction and per lesion). The unpaired *T*-test was executed for normally distributed data and the Mann-Whitney *U* test for nonparametric data. Additionally, the systematic error was quantified as the sd of the mean per lesion, and the random error was quantified as the root mean square of the sd per lesion. Spearman correlation analysis was used to investigate the relation between time and motion. A Kruskal Wallis Test was utilized to investigate the relationship of lesion location (six pelvic areas) and lesion volume (≤1.4 cc or >1.4 cc (mean volume)) with lesion displacement.

## Results

A total of 56 lesions were evaluated for this analysis and data of 11 lesions were not included due to either lack of sufficient number of post-fraction CBCTs (<3 CBCTs, n = 2) or a poor to very poor visibility on CBCT (n = 9). Therefore, 45 lesions in 37 patients were included in this analysis (Table 1).

**Table 1:** Characteristics of lesions included in this analysis (n = 45).

**Characteristics**	**N (%)**
Lesions per patient	
1	30 (81 %)
2	6 (16 %)
3	1 (3 %)

Location	
Internal iliac	13 (29 %)
External iliac	16 (36 %)
Common iliac	4 (9 %)
Obturator	3 (7 %)
Para-rectal	5 (11 %)
Presacral	4 (9 %)

Lateralisation	
Left	24 (53 %)
Right	17 (38 %)
Central	4 (9 %)

Lesion volume (median (range))	0.8 (0.2–10.4) cm^3^
Fraction time (mean (sd))	8.7 (2.7) minutes

Visibility score	
Excellent	15 (33 %)
Good	20 (44 %)
Average	10 (22 %)

All lymph node lesions were located in the pelvis and most lesions were located along the iliac vessels (n = 33, 74 %) (Table 1). In 7 patients more than one pelvic lymph node was treated, which comprised a total of 15 lesions (1 patient 3 lesions, 6 patients 2 lesions). Therefore, 37 unique planning CTs, 194 pre-fraction CBCTs and 188 post-fraction CBCTs were included in this analysis, with 45, 224, and 216 GTVs delineated, respectively.

Mean (sd) fraction time from start of pre-fraction CBCT to start of post-fraction CBCT was 8.7 (2.7) minutes. The GTV volume on the planning CT scan showed a range of 0.2 cm^3^ to 10.4 cm^3^ (median 0.8 cm^3^). On CBCT, the mean (sd) GTV volume was 73 % (17 %) of the volume observed on the planning CT. The mean GTV volume during fractions 1 to 5 were 1.1 cm^3^, 1.1 cm^3^, 0.9 cm^3^, 0.9 cm^3^, and 0.8 cm^3^, respectively. A strong negative correlation was observed between the number of executed fractions and mean GTV volume per fraction (rs = −0.303, p < 0.001), indicating a statistically significant decrease in lesion volume during radiotherapy treatment.

**Fig. 1:**
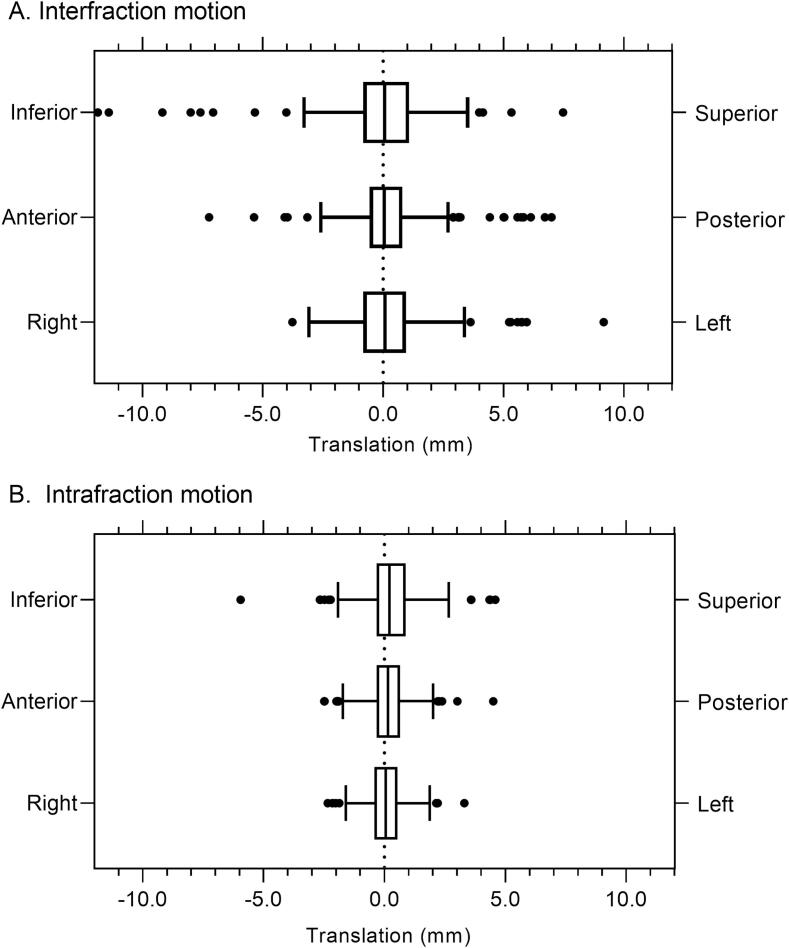
**Lesion inter- and intrafraction motion in three directions.**Tukey method boxplots: Box with interquartile range (IQR), Whiskers from median to 25th percentile minus 1.5*IQR and median to 75th percentile plus 1.5*IQR.

Interfraction motion analysis was based on 45 planning CT and 224 pre-fraction GTV delineations. The mean (sd) lesion interfraction motion was 0.2 (1.6) mm LR, 0.3 (1.8) mm AP, and −0.1 (2.2) mm SI and the SI direction showed the largest systematic and random errors (Table 2). The mean (sd) 3D vector interfraction motion was 2.5 (2.1) mm and in 95 % of fractions the mean interfraction vector was ≤6.8 mm (Fig. 1).

**Table 2:** Inter- and intrafraction lesion motion per fraction.

	Left – Right (mm)	Anterior – Posterior (mm)	Superior – Inferior (mm)	3D Vector (mm)
Interfraction motion				
Mean	0.2	0.2	−0.1	2.5
Sd	1.6	1.8	2.2	2.1
**Systematic error**	**1.4**	**1.4**	**1.7**	**1.8**
**Random error**	**0.8**	**1.1**	**1.3**	**1.2**
Min	−3.8	−7.2	−11.9	0.2
Max	9.2	7.0	7.5	15.6
95th percentile	3.0	4.4	4.0	6.8
>5 mm, N (%)	7 (3.1)	9 (4.0)	9 (4.0)	
>3 mm, N (%)	11 (4.9)	17 (7.6)	19 (8.4)	

Intrafraction motion				
Mean	0.1	0.1	0.3	1.5
Sd	0.8	0.9	1.1	0.9
**Systematic error**	**0.5**	**0.6**	**0.5**	**0.5**
**Random error**	**0.7**	**0.7**	**1.0**	**0.8**
Min	−2.4	−2.5	−6.0	0.2
Max	3.3	4.5	4.6	6.1
95th percentile	1.7	1.9	2.5	3.0
>5 mm, N (%)	0 (0)	0 (0)	1 (0.4)	
>3 mm, N (%)	1 (0.4)	2 (0.9)	6 (2.6)

For intrafraction motion, analysis was based on 216 GTVs delineated on both pre-fraction and post-fraction CBCT. The mean (sd) lesion intrafraction motion was 0.1 (0.8) mm LR, 0.1 (0.9) mm AP, and 0.3 (1.1) mm SI (Fig. 1). The mean (sd) 3D intrafraction lesion vector was 1.5 (0.9) mm and in 95 % of fractions the mean intrafraction vector was ≤3.0 mm (Table 2). We found no correlation between intrafraction motion (LR, SI and AP) and fraction time (Appendix A.1).

**Fig. 2:**
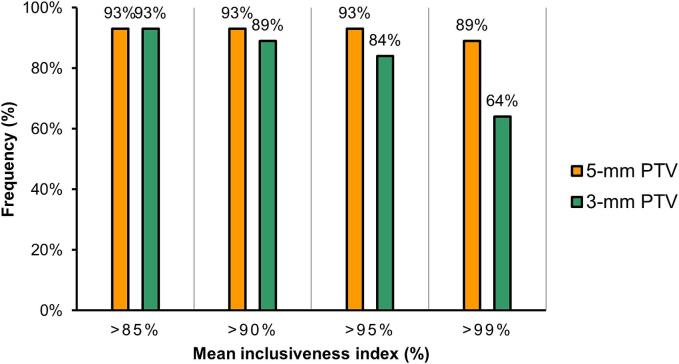
Frequency of mean inclusiveness index values per lesion for a margin of 5 mm and a margin of 3 mm.

The addition of a PTV margin of 5.0 and 3.0 mm to the planning GTV resulted in a corresponding median PTV volume of 3.0 cc and 5.6 cc, respectively. The mean (sd) inclusiveness index for the PTV with a 5-mm margin was 98.5 % (8.0 %) per fraction and 98.5 % (5.3 %) per lesion. The mean (sd) inclusiveness index for the 3-mm margin was 96.4 % (11.9 %) per fraction and 96.4 % (8.9 %) per lesion. A mean inclusiveness of less than 95 % by the 5-mm PTV was observed in only 3 lesions (7 %), which were located presacral (0.4 cm^3^), and two lesions para-rectal (1.3 cm^3^ and 4.2 cm^3^) (Fig. 2). The inclusiveness indices of these lesions were 81 %, 85 % and 75 %, respectively.

The inclusiveness index was identical to the treatment planning coverage in 37/45 lesions (82 %). A complete report of the inclusiveness index and coverage (V33.25 Gy) per lesion is presented in Appendix A.2.

**Fig. 3:**
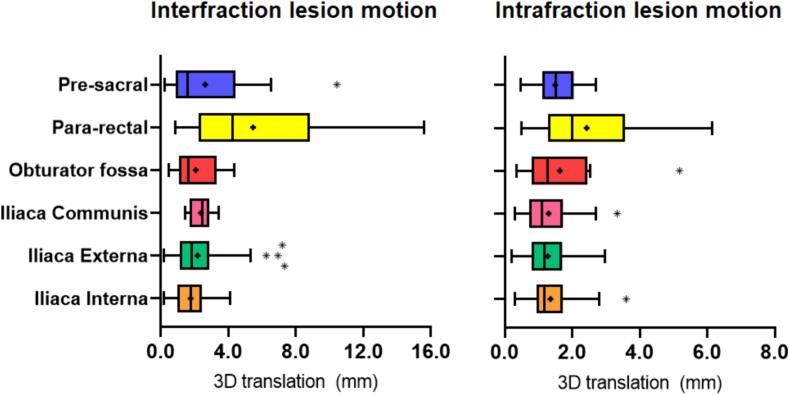
**Inter- and intrafraction lesion motion per location in the pelvis.**Tukey method boxplots representing the interfraction and intrafraction motion per lesion location. Box with interquartile range (IQR), Whiskers from median to 25th percentile minus 1.5*IQR and median to 75th percentile plus 1.5I*QR, + indicating mean. The Kruskal-Wallis H test indicated a significant difference in the mean inter- and intrafraction lesion motion among the lesion locations. (p < 0.001 and p0.007).

The mean interfraction lesion motion (3D vector) varied significantly among the pelvic lesion locations (p < 0.001), and para-rectal locations (n = 5, 11 %) were associated with a significantly higher mean interfraction motion compared to other pelvic locations (mean 5.5 vs. 2.1 mm). Similarly, for intrafraction motion, the lesion motion was significantly higher for para-rectal lesions compared to other lesions (mean 2.4 vs. 1.4 mm) (Fig. 3 and Appendix A.3).

Interfraction lesion motion was significantly higher for lesions with volume >1.4 cc (N = 10) compared to lesions ≤1.4 cc (mean vector 3.5 vs. 2.2 mm, p0.002) while intrafraction motion was significantly lower for lesions with volume >1.4 cc (mean vector 1.3 mm vs. 1.5 mm, p0.01).

As a consequence, the inclusiveness index for lesions with a volume >1.4 cc was significantly lower for both the 5.0-mm margin (97 % vs. 99 %) and the 3.0-mm margin (94 % vs. 99 %) compared to lesions with a volume of ≤1.4 cc. (Appendix A.3).

## Discussion

This analysis showed that intrafraction motion of oligometastatic pelvic lymph nodes was limited to 3 mm in 97–100 % of fractions and the mean intrafraction motion was near zero in all directions (mean 0.1–0.3 mm). The use of a PTV margin of 5 mm resulted in a mean lesion inclusiveness index of 99 % (±5 %), and a PTV margin of 3 mm in a mean of 96 % (±9 %). The relatively short overall treatment time on CBCT-linac (mean 8.7 min) did not correlate with an increase in intrafraction motion.

We found that lymph node lesions maintained a stable position throughout SBRT treatment since systematic errors in intrafraction motion were low, ranging from 0.5 to 0.6 mm in all three directions. These results are in line with systematic errors (range 0.3–1.0 mm) reported for MR-guided para-aortal and pelvic lymph node SBRT during the first 15–17 min interval. However, systematic errors were larger throughout the full MR-guided treatment with a mean treatment time interval >30 min [11]. Likewise, another MR-linac based study reported an increase in motion over time during lymph node treatment [15]. Consequently, the fast treatment facilitated by CBCT-linac is advantageable, not only to save resources and contribute to patient convenience but also to limit intrafraction motion [16].

Other research on pelvic lymph node intrafraction motion is limited. Two studies reporting on lymph node intrafraction motion have focused on mediastinal lymph nodes and reported systematically larger superior motion related to respiration [18], [19]. Yet, in both elective pelvic field radiotherapy and prostate SBRT, larger mobility was reported for the AP direction [14], [20], [21], [22]. Importantly, we found that mean lymph node intrafraction motion was near zero in all directions and systematic errors were equally distributed, which affirms the application of a uniform (isotropic) margin for CBCT-guided SBRT to pelvic lymph nodes.

In this study, pre- and post-fraction imaging were used to assess intrafraction motion, which could underestimate lesion shifts during treatment. Nevertheless, pelvic nodal tracking on cine MR imaging demonstrated comparable coverage and maximum lesion motion compared to studies using pre- and post-fraction MRI [11], [15], [16]. These results highlight the reliability of the commonly applied method to evaluate intrafraction motion by using pre- and post-fraction imaging and support the robustness of our results.

The maximum interfraction motion in this report ranged from −11.9 mm to 7.5 mm, which is twice as large as the observed intrafraction motion. However, in image-guided SBRT, correction of interfraction lesion displacement is already addressed through pre-fraction imaging, which would result in limited impact of this interfraction motion on lesion coverage. Image-guided interfraction motion correction depends on lesion visibility since, within our institute, the visual check of lesion alignment on CBCT is the basis of manual correction before SBRT. Lesion visibility has been mentioned as a potential limitation of CBCT-guided SBRT compared to MRI-guided SBRT, and our data could be useful to determine the margin necessary for treatment of lesions with limited visibility [23]. Reassuringly, only nine lesions required exclusion from this analysis due to limited visualization on CBCT. The continuous improvements in CBCT image quality contribute to adequate visibility, allowing the clear identification of pelvic metastases included in this study.

The application of an isotropic margin of 3 mm resulted in a high mean lesion inclusiveness index of 96 %, keeping in mind that the index does not consider interfraction correction and other treatment-related uncertainties. The presented inclusiveness index offers the advantage of being irrespective of the planning method, making it applicable to any institution, yet it does not take dose-fall off into account. A comparison of the inclusiveness index with our treatment planning coverage (V3325cGy) revealed that the index matched the planned coverage in 82 % of lesions and in only 7 % of lesions the coverage was overestimated by a maximum coverage difference of 5 %. Hence, these encouraging results of the inclusiveness index coverage provide the opportunity for a safe margin reduction to 3 mm if clinically necessary.

In this study, lesion motion varied significantly among different pelvic lesion locations. This difference in lesion motion according to location within the pelvis has been previously mentioned for whole pelvis radiotherapy based on repetitive MRI [14]. Specifically, we found that para-rectal lesions (n = 5, 11 %) were associated with >50 % higher mean translational motion compared to other lesion locations. This larger translational motion demonstrated in our study is in line with motion results reported for para-rectal lymph node metastases of rectal carcinoma, which showed large nodal mobility and a mean intrafraction motion ranging from −0.2 mm to 0.7 mm, comparable to our results of −0.3–0.6 mm in all directions [24]. This larger translational motion for para-rectal nodes may need a more specific approach with the consideration of an adapted PTV margin or the use of MR-guided beam gating to limit the chance of geographical miss during treatment.

A limitation of this study is the uncertainty due to possible delineation inaccuracy and CBCT slice thickness, which could introduce bias in SI displacements. Nevertheless, with the aim to limit possible inter-observer variation in delineation, we appointed one physician to delineate all lesions on both planning CT and CBCTs. Moreover, the effect of multiple lesions (GTVs) within one patient was out of scope for this study, while multiple lesions in one patient could require a different treatment strategy considering margins and verification [16].

Knowledge of lesion motion during SBRT is crucial to evaluate margin application and guarantee the accuracy of treatment delivery [3], [4], [8]. Unfortunately, published data on pelvic lymph node motion during CBCT-guided SBRT are lacking, while SBRT aimed at oligometastatic pelvic lymph node metastases is increasingly applied. This comprehensive report on both inter- and intrafraction lesion motion provided extensive results and included a representative number (N = 45) of prostate cancer lymph node metastases located in various areas of the pelvis and incorporated a variety of lesion volumes. Hereby, these results provide an indispensable insight into pelvic lymph node motion and robustness of currently used PTV margins for CBCT-guided SBRT. Moreover, we identified that margins for individual pelvic lymph nodes can be differentiated according to their anatomical location within the pelvis, potentially providing the opportunity to reduce the margin to at least 3 mm in most pelvic regions.

## Conclusions

This analysis of pelvic lymph node motion during CBCT-guided stereotactic radiotherapy demonstrated a limited inter- and intrafraction motion supporting the use of the clinically applied 5 mm PTV margin and offering an opportunity to reduce this margin to 3 mm for most pelvic lymph node metastases. Importantly, the location of pelvic lymph node metastases was a significant factor influencing lesion motion and, in case of a para-rectal localization, application of a larger margin or MR-guided beam gating should be considered.

## CRediT authorship contribution statement

**J. Janssen:** Conceptualization, Methodology, Formal analysis, Investigation, Writing – original draft, Writing – review & editing, Visualization, Project administration. **F.H.E. Staal:** Methodology, Writing – original draft, Writing – review & editing. **J.A. Langendijk:** Resources, Writing – review & editing, Supervision. **S. Both:** Resources, Writing – review & editing. **C.L. Brouwer:** Conceptualization, Methodology, Writing – review & editing, Supervision. **S. Aluwini:** Conceptualization, Methodology, Writing – original draft, Writing – review & editing, Supervision.

## Declaration of competing interest

The authors declare the following financial interests/personal relationships which may be considered as potential competing interests: The ADOPT clinical trial (Principal Investigator S. Aluwini, NCT04302454) has been funded by the Dutch Cancer Society (KWF, Amsterdam, Grant number 12448). The department of Radiation Oncology of the University Medical Center Groningen has research contracts with IBA, RaySearch, Siemens, Elekta, Leoni, and Mirada, and has received grants from the Dutch Cancer Society and the European Union.

## References

[1] Zilli T., Achard V., Dal Pra A., Schmidt-Hegemann N., Jereczek-Fossa B.A., Lancia A. (2022). Recommendations for radiation therapy in oligometastatic prostate cancer: An ESTRO-ACROP Delphi consensus. Radiother Oncol.

[2] Palma D.A., Olson R., Harrow S., Gaede S., Louie A.V., Haasbeek C. (2019). Stereotactic ablative radiotherapy versus standard of care palliative treatment in patients with oligometastatic cancers (SABR-COMET): a randomised, phase 2, open-label trial. Lancet.

[3] Rogowski P., Roach M., Schmidt-Hegemann N.S., Trapp C., von Bestenbostel R., Shi R. (2021). Radiotherapy of oligometastatic prostate cancer: a systematic review. Radiat Oncol.

[4] Lawal I.O., Lengana T., Popoola G.O., Orunmuyi A.T., Kgatle M.M., Mokoala K.M.G. (2021). Pattern of Prostate cancer recurrence assessed by (68)Ga-PSMA-11 PET/CT in men treated with primary local therapy. J Clin Med.

[5] Palma D.A., Haasbeek C.J., Rodrigues G.B., Dahele M., Lock M., Yaremko B. (2012). Stereotactic ablative radiotherapy for comprehensive treatment of oligometastatic tumors (SABR-COMET): study protocol for a randomized phase II trial. BMC Cancer.

[6] Decaestecker K., De Meerleer G., Ameye F., Fonteyne V., Lambert B., Joniau S. (2014). Surveillance or metastasis-directed therapy for OligoMetastatic Prostate cancer recurrence (STOMP): study protocol for a randomized phase II trial. BMC Cancer.

[7] Radwan N., Phillips R., Ross A., Rowe S.P., Gorin M.A., Antonarakis E.S. (2017). A phase II randomized trial of Observation versus stereotactic ablative RadiatIon for OLigometastatic prostate CancEr (ORIOLE). BMC Cancer.

[8] Benedict S.H., Yenice K.M., Followill D., Galvin J.M., Hinson W., Kavanagh B. (2010). Stereotactic body radiation therapy: the report of AAPM Task Group 101. Med Phys.

[9] van Herk M., Remeijer P., Rasch C., Lebesque J.V. (2000). The probability of correct target dosage: dose-population histograms for deriving treatment margins in radiotherapy. Int J Radiat Oncol Biol Phys.

[10] Lievens Y., Guckenberger M., Gomez D., Hoyer M., Iyengar P., Kindts I. (2020). Defining oligometastatic disease from a radiation oncology perspective: an ESTRO-ASTRO consensus document. Radiother Oncol.

[11] Werensteijn-Honingh A.M., Jurgenliemk-Schulz I.M., Gadellaa-Van Hooijdonk C.G., Sikkes G.G., Vissers N., Winkel D. (2021). Impact of a vacuum cushion on intrafraction motion during online adaptive MR-guided SBRT for pelvic and para-aortic lymph node oligometastases. Radiother Oncol.

[12] Janssen J., Brouwer C.L., Staal F.H.E., van Herpt H.E., Both S., Langendijk J.A. (2023). Simple immobilization for stereotactic radiotherapy aimed at pelvic metastases. Phys Imaging Radiat Oncol.

[13] La Fauci F., Augugliaro M., Mazzola G.C., Comi S., Pepa M., Zaffaroni M. (2022). Dosimetric evaluation of the inter-fraction motion of organs at risk in SBRT for nodal oligometastatic prostate cancer. Appl Sci-Basel.

[14] Bjoreland U., Jonsson J., Alm M., Beckman L., Nyholm T., Thellenberg-Karlsson C. (2018). Inter-fraction movements of the prostate and pelvic lymph nodes during IGRT. J Radiat Oncol.

[15] Snyder J., Smith B., St-Aubin J., Dunkerley D., Shepard A., Caster J. (2023). Intra-fraction motion of pelvic oligometastases and feasibility of PTV margin reduction using MRI guided adaptive radiotherapy. Front Oncol.

[16] Winkel D., Bol G.H., Werensteijn-Honingh A.M., Intven M.P.W., Eppinga W.S.C., Hes J. (2020). Target coverage and dose criteria based evaluation of the first clinical 1.5T MR-linac SBRT treatments of lymph node oligometastases compared with conventional CBCT-linac treatment. Radiother Oncol.

[17] Janssen J., Staal F.H.E., Brouwer C.L., Langendijk J.A., de Jong I.J., van Moorselaar R.J.A. (2022). Androgen Deprivation therapy for Oligo-recurrent Prostate cancer in addition to radioTherapy (ADOPT): study protocol for a randomised phase III trial. BMC Cancer.

[18] Schmidt M.L., Hoffmann L., Moller D.S., Knap M.M., Rasmussen T.R., Folkersen B.H. (2018). Systematic intrafraction shifts of mediastinal lymph node targets between setup imaging and radiation treatment delivery in lung cancer patients. Radiother Oncol.

[19] Pantarotto J.R., Piet A.H., Vincent A., van Sornsen de Koste J.R., Senan S. (2009). Motion analysis of 100 mediastinal lymph nodes: potential pitfalls in treatment planning and adaptive strategies. Int J Radiat Oncol Biol Phys.

[20] Lovelock D.M., Messineo A.P., Cox B.W., Kollmeier M.A., Zelefsky M.J. (2015). Continuous monitoring and intrafraction target position correction during treatment improves target coverage for patients undergoing SBRT prostate therapy. Int J Radiat Oncol Biol Phys.

[21] Panizza D., Faccenda V., Lucchini R., Daniotti M.C., Trivellato S., Caricato P. (2022). Intrafraction prostate motion management during dose-escalated linac-based stereotactic body radiation therapy. Front Oncol.

[22] Lawes R., Carter E., Hussein M., Murray J., McNair H.A. (2021). Retrospective audit of inter-fraction motion for pelvic node radiotherapy in prostate cancer patients. Radiography (Lond).

[23] Noel C.E., Parikh P.J., Spencer C.R., Green O.L., Hu Y., Mutic S. (2015). Comparison of onboard low-field magnetic resonance imaging versus onboard computed tomography for anatomy visualization in radiotherapy. Acta Oncol.

[24] Kensen C.M., Betgen A., Wiersema L., Peters F.P., Kayembe M.T., Marijnen C.A.M. (2023). Online adaptive MRI-guided radiotherapy for primary tumor and lymph node boosting in rectal cancer. Cancers (Basel).

