# Supplementary material for: Pelvic lymph node motion during cone-beam computed tomography guided stereotactic radiotherapy
Source: Clin Transl Radiat Oncol. 2024 May 11;47:100794. doi: 10.1016/j.ctro.2024.100794 (PMC11127188; doi:10.1016/j.ctro.2024.100794)
Supplement: Supplementary Data 1 [file mmc1.docx]

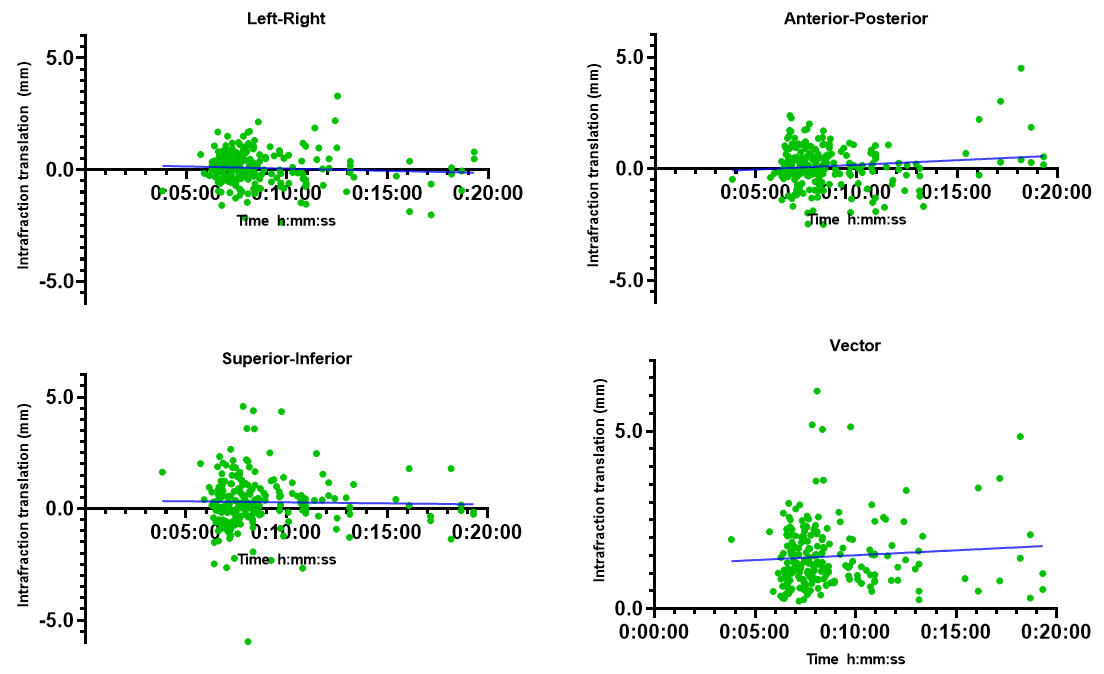


Scatterplots representing the correlation between lesion intrafraction translation and treatment time. The blue line is the best fitted line.
