# Supplementary material for: Pelvic lymph node motion during cone-beam computed tomography guided stereotactic radiotherapy
Source: Clin Transl Radiat Oncol. 2024 May 11;47:100794. doi: 10.1016/j.ctro.2024.100794 (PMC11127188; doi:10.1016/j.ctro.2024.100794)
Supplement: Supplementary Data 2 [file mmc2.docx]

| **Lesion** | **Location** | **GTV Volume** | **Remarks***** | **Variable** | **Fx1** | **Fx2** | **Fx3** | **Fx4** | **Fx5** | **Mean** | **∆** |
| --- | --- | --- | --- | --- | --- | --- | --- | --- | --- | --- | --- |
| 1 | External iliac | 3.2 cm3 |  | Inclusiveness index (%) | 100 | 100 | 98 | 98 | 99 | **99** |  |
|  |  |  |  | V33.25Gy (%) | 100 | 100 | 100 | 100 | 100 | **100** | **-1** |
| 2 | Internal iliac | 2.9 cm3 | shared CBCT & separate plan [2, 3] | Inclusiveness index (%) | 100 | 100 | 100 | 100 | 100 | **100** |  |
|  |  |  |  | V33.25Gy (%) | 100 | 100 | 100 | 100 | 100 | **100** | **0** |
| 3 | Para-rectal | 1.1 cm3 | shared CBCT & separate plan [2, 3] | Inclusiveness index (%) | 100 | 100 | 100 | 100 | 100 | **100** |  |
|  |  |  |  | V33.25Gy (%) | 100 | 100 | 100 | 100 | 100 | **100** | **0** |
| 4 | Obturator | 0.5 cm3 |  | Inclusiveness index (%) | 100 | 100 | 100 | 100 | 100 | **100** |  |
|  |  |  |  | V33.25Gy (%) | 100 | 100 | 100 | 100 | 100 | **100** | **0** |
| 5 | External iliac | 0.5 cm3 | shared CBCT & plan [5, 6, 7] | Inclusiveness index (%) | 100 | 100 | 100 | 100 | 100 | **100** |  |
|  |  |  |  | V33.25Gy (%) | 100 | 100 | 100 | 100 | 100 | **100** | **0** |
| 6 | Presacral | 0.4 cm3 | shared CBCT & plan [5, 6, 7] | Inclusiveness index (%) | 100 | 100 | 100 | 100 | 100 | **100** |  |
|  |  |  |  | V33.25Gy (%) | 100 | 100 | 100 | 100 | 100 | **100** | **0** |
| 7 | External iliac | 0.6 cm3 | shared CBCT & plan [5, 6, 7] | Inclusiveness index (%) | 100 | 100 | 100 | 100 | 100 | **100** |  |
|  |  |  |  | V33.25Gy (%) | 100 | 100 | 100 | 100 | 100 | **100** | **0** |
| 8 | External iliac | 0.6 cm3 |  | Inclusiveness index (%) | 100 | 100 | 100 | 100 | 100 | **100** |  |
|  |  |  |  | V33.25Gy (%) | 100 | 100 | 100 | 100 | 100 | **100** | **0** |
| 9 | Common iliac | 0.5 cm3 |  | Inclusiveness index (%) | 100 | 100 | 100 | 100 | 100 | **100** |  |
|  |  |  |  | V33.25Gy (%) | 100 | 100 | 100 | 100 | 100 | **100** | **0** |
| 10 | Internal iliac | 0.5 cm3 | shared CBCT & plan [10, 11] | Inclusiveness index (%) | 100 | 100 | 100 | 100 | 100 | **100** |  |
|  |  |  |  | V33.25Gy (%) | 100 | 100 | 100 | 100 | 100 | **100** | **0** |
| 11 | External iliac | 0.8 cm3 | shared CBCT & plan [10, 11] | Inclusiveness index (%) | 100 | 100 | 100 | 100 | 100 | **100** |  |
|  |  |  |  | V33.25Gy (%) | 100 | 100 | 100 | 100 | 100 | **100** | **0** |
| 12 | Internal iliac | 1.0 cm3 |  | Inclusiveness index (%) | 100 | 100 | 100 | 100 | 100 | **100** |  |
|  |  |  |  | V33.25Gy (%) | 100 | 100 | 100 | 100 | 100 | **100** | **0** |
| 13 | External iliac | 0.5 cm3 |  | Inclusiveness index (%) | 100 | 100 | 100 | 100 | 100 | **100** |  |
|  |  |  |  | V33.25Gy (%) | 100 | 100 | 100 | 100 | 100 | **100** | **0** |
| 14 | Internal iliac | 0.2 cm3 |  | Inclusiveness index (%) | 100 | 100 | 100 | 100 | 100 | **100** |  |
|  |  |  |  | V33.25Gy (%) | 100 | 100 | 100 | 100 | 100 | **100** | **0** |
| 15 | External iliac | 0.4 cm3 |  | Inclusiveness index (%) | 100 | 100 | 100 | 100 | 100 | **100** |  |
|  |  |  |  | V33.25Gy (%) | 100 | 100 | 100 | 100 | 100 | **100** | **0** |
| 16 | Internal iliac | 0.4 cm3 |  | Inclusiveness index (%) | 100 | 100 | 100 | 100 | 100* | **100** |  |
|  |  |  |  | V33.25Gy (%) | 100 | 100 | 100 | 100 | 100* | **100** | **0** |
| 17 | External iliac | 4.0 cm3 | shared CBCT & plan [17, 18] | Inclusiveness index (%) | 95* | 92 | 100* | 100 | 97 | **97** |  |
|  |  |  |  | V33.25Gy (%) | 100* | 99 | 100* | 100 | 100 | **100** | **-3** |
| 18 | External iliac | 0.5 cm3 | shared CBCT & plan [17, 18] | Inclusiveness index (%) | 100* | 100 | 100* | 100 | 100 | **100** |  |
|  |  |  |  | V33.25Gy (%) | 100* | 100 | 100* | 100 | 100 | **100** | **0** |
| 19 | External iliac | 0.3 cm3 |  | Inclusiveness index (%) | 100 | 100 | 100 | 100 | 100 | **100** |  |
|  |  |  |  | V33.25Gy (%) | 100 | 100 | 87 | 97 | 92 | **95** | **5** |
| 20 | External iliac | 0.7 cm3 |  | Inclusiveness index (%) | 100 | 100* | 100 | 99 | 100 | **100** |  |
|  |  |  |  | V33.25Gy (%) | 100 | 100* | 100 | 100 | 100 | **100** | **0** |
| 21 | External iliac | 1.2 cm3 |  | Inclusiveness index (%) | 100 | 100 | 100 | 100 | 100 | **100** |  |
|  |  |  |  | V33.25Gy (%) | 100 | 100 | 100 | 100 | 100 | **100** | **0** |
| 22 | Internal iliac | 0.4 cm3 |  | Inclusiveness index (%) | 100 | 100 | 99 | 100 | 100 | **100** |  |
|  |  |  |  | V33.25Gy (%) | 100 | 100 | 100 | 100 | 100 | **100** | **0** |
| 23 | External iliac | 0.9 cm3 |  | Inclusiveness index (%) | 100 | 100 | 100 | 100 | 100 | **100** |  |
|  |  |  |  | V33.25Gy (%) | 100 | 100 | 100 | 100 | 100 | **100** | **0** |
| 24 | Presacral | 0.4 cm3 |  | Inclusiveness index (%) | 100 | 78 | 100* | 25 | 100 | **81** |  |
|  |  |  |  | V33.25Gy (%) | 100 | 100 | 100* | 59 | 100 | **92** | **-11** |
| 25 | Internal iliac | 0.8 cm3 | shared CBCT & plan [25, 26] | Inclusiveness index (%) | 100 | 100 | 100 | 100 | 100 | **100** |  |
|  |  |  |  | V33.25Gy (%) | 100 | 100 | 100 | 100 | 100 | **100** | **0** |
| 26 | Internal iliac | 0.2 cm3 | shared CBCT & plan [25, 26] | Inclusiveness index (%) | 100 | 100 | 100 | 100 | 100 | **100** |  |
|  |  |  |  | V33.25Gy (%) | 100 | 100 | 100 | 100 | 100 | **100** | **0** |
| 27 | External iliac | 4.8 cm3 |  | Inclusiveness index (%) | 100 | 100 | 100 | 100 | 100 | **100** |  |
|  |  |  |  | V33.25Gy (%) | 100 | 100 | 100 | 100 | 100 | **100** | **0** |
| 28 | External iliac | 0.8 cm3 |  | Inclusiveness index (%) | 100 | 100 | 100 | 100 | 100 | **100** |  |
|  |  |  |  | V33.25Gy (%) | 100 | 100 | 100 | 100 | 100 | **100** | **0** |
| 29 | External iliac | 0.8 cm3 | separate CBCT & plan [29, 30] | Inclusiveness index (%) | 100 | 100 | 100 | 100 | 100 | **100** |  |
|  |  |  |  | V33.25Gy (%) | 100 | 100 | 100 | 100 | 100 | **100** | **0** |
| 30 | Internal iliac | 0.8 cm3 | separate CBCT & plan [29, 30] | Inclusiveness index (%) | 100 | 100 | 100 | 100 | 100 | **100** |  |
|  |  |  |  | V33.25Gy (%) | 100 | 100 | 100 | 100 | 100 | **100** | **0** |
| 31 | Para-rectal | 0.3 cm3 | separate CBCT & plan [32, 33] | Inclusiveness index (%) | 100 | 100 | 100 | 100 | 100 | **100** |  |
|  |  |  |  | V33.25Gy (%) | 100 | 100 | 100 | 100 | 100 | **100** | **0** |
| 32 | Presacral | 0.9 cm3 | separate CBCT & plan [32, 33] | Inclusiveness index (%) | 100 | 100 | 100 | 100 | 100 | **100** |  |
|  |  |  |  | V33.25Gy (%) | 100 | 100 | 100 | 100 | 100 | **100** | **0** |
| 33 | Common iliac | 1.5 cm3 |  | Inclusiveness index (%) | 100 | 100 | 100 | 100 | 100 | **100** |  |
|  |  |  |  | V33.25Gy (%) | 100 | 100 | 100 | 100 | 100 | **100** | **0** |
| 34 | Para-rectal | 10.4 cm3 |  | Inclusiveness index (%) | 100 | 100 | ** | 100 | 100 | **100** |  |
|  |  |  |  | V33.25Gy (%) | 99 | 99 | ** | 100 | 99 | **99** | **1** |
| 35 | Para-rectal | 1.3 cm3 |  | Inclusiveness index (%) | 100 | 100 | 59 | 95 | 69 | **85** |  |
|  |  |  |  | V33.25Gy (%) | 100 | 100 | 86 | 100 | 93 | **96** | **-11** |
| 36 | Internal iliac | 0.5 cm3 |  | Inclusiveness index (%) | 100 | 100 | 100 | 100 | 100 | **100** |  |
|  |  |  |  | V33.25Gy (%) | 100 | 100 | 100 | 100 | 100 | **100** | **0** |
| 37 | Obturator | 0.3 cm3 |  | Inclusiveness index (%) | 89 | 100 | 100 | 100 | 100 | **98** |  |
|  |  |  |  | V33.25Gy (%) | 100 | 100 | 100 | 100 | 100 | **100** | **-3** |
| 38 | Internal iliac | 1.5 cm3 |  | Inclusiveness index (%) | 100 | 100 | 100 | 100 | 100 | **100** |  |
|  |  |  |  | V33.25Gy (%) | 100 | 100 | 100 | 100 | 100 | **100** | **0** |
| 39 | Internal iliac | 5.3 cm3 |  | Inclusiveness index (%) | 100 | 100* | 100 | 100 | 100 | **100** |  |
|  |  |  |  | V33.25Gy (%) | 100 | 100* | 100 | 100 | 100 | **100** | **0** |
| 40 | Common iliac | 0.8 cm3 |  | Inclusiveness index (%) | 100 | 100 | 100 | 100 | 100 | **100** |  |
|  |  |  |  | V33.25Gy (%) | 100 | 100 | 100 | 100 | 100 | **100** | **0** |
| 41 | Para-rectal | 4.2 cm3 |  | Inclusiveness index (%) | 98 | 36 | 82 | 67 | 92 | **75** |  |
|  |  |  |  | V33.25Gy (%) | 100 | 42 | 72 | 54 | 87 | **71** | **4** |
| 42 | Common iliac | 2.8 cm3 |  | Inclusiveness index (%) | 100 | 100 | 100 | 100 | 100 | **100** |  |
|  |  |  |  | V33.25Gy (%) | 100 | 100 | 100 | 100 | 100 | **100** | **0** |
| 43 | Presacral | 0.8 cm3 |  | Inclusiveness index (%) | 100 | 100 | 100 | 100 | 100 | **100** |  |
|  |  |  |  | V33.25Gy (%) | 100 | 100 | 100 | 100 | 100 | **100** | **0** |
| 44 | Internal iliac | 0.3 cm3 |  | Inclusiveness index (%) | 100 | 100 | 100 | 100 | 100 | **100** |  |
|  |  |  |  | V33.25Gy (%) | 100 | 100 | 100 | 100 | 100 | **100** | **0** |
| 45 | Obturator | 1.0 cm3 |  | Inclusiveness index (%) | 100 | 100 | 100 | 100 | 100 | **100** |  |
|  |  |  |  | V33.25Gy (%) | 100 | 100 | 100 | 100 | 100 | **100** | **0** |

*Post-CBCT missing, value based on pre-fraction CBCT result
**CBCT of this fraction not included, visibility rating very poor
*** Remark added in case of multiple lesions in one patient

| **∆ mean** | **Frequency (%)** | **Min/Max value** |
| --- | --- | --- |
| Negative (Inclusiveness underestimating coverage) | 5 (11%) | -11% |
| 0 | 37 (82%) |  |
| Positive (Inclusiveness overestimating coverage) | 3 (7%) | 5% |
