# Supplementary material for: Pelvic lymph node motion during cone-beam computed tomography guided stereotactic radiotherapy
Source: Clin Transl Radiat Oncol. 2024 May 11;47:100794. doi: 10.1016/j.ctro.2024.100794 (PMC11127188; doi:10.1016/j.ctro.2024.100794)
Supplement: Supplementary Data 3 [file mmc3.docx]

**Table C.1**

|  | **Para-rectal** | **Other locations** | **P value** |
| --- | --- | --- | --- |
| Interfraction motion (mean (sd)) | | | |
| LR (mm) | 2.2 (2.9) | 0.0 (1.2) | **0.001** |
| AP (mm) | -0.2 (2.2) | 0.3 (1.7) | 0.197 |
| SI (mm) | -2.1 (4.9) | 0.2 (1.4) | **0.036** |
| 3D (mm) | 5.5 (4.0) | 2.1 (1.4) | **<0.001** |
| Intrafraction motion (mean (sd)) | | | |
| LR (mm) | -0.3 (0.9) | 0.12 (0.7) | **0.015** |
| AP (mm) | 0.12 (1.7) | 0.12 (0.8) | 0.992 |
| SI (mm) | 0.6 (2.1) | 0.3 (1.0) | 0.460 |
| 3D (mm) | 2.4 (1.6) | 1.4 (0.7) | **<0.001** |

**Table C.2**

|  | **Vol <=1.4** | **Vol >1.4** | **P value** |
| --- | --- | --- | --- |
| Interfraction motion (mean (sd)) | | | |
| LR (mm) | 0.2 (1.4) | 0.2 (2.3) | 0.909 |
| AP (mm) | 0.1 (1.5) | 0.8 (2.4) | 0.05 |
| SI (mm) | 0.1 (1.8) | -0.5 (3.2) | 0.203 |
| 3D (mm) | 2.2 (1.7) | 3.5 (3.2) | **0.005** |
| Intrafraction motion (mean (sd)) | | | |
| LR (mm) | 0.1 (0.8) | 0.2 (0.7) | 0.375 |
| AP (mm) | 0.1 (1.0) | 0.1 (0.7) | 0.935 |
| SI (mm) | 0.3 (1.1) | 0.1 (1.4) | 0.261 |
| 3D (mm) | 1.5 (0.9) | 1.3 (1.1) | **0.02** |

Unpaired T-test for LR/AP/SI data and MannWhitney for 3D vector data. In case of unpaired T-test the equality of variances was tested by Levene’s test.
